# Transactions of the Medical Society of the State of New York

**Published:** 1841-05-22

**Authors:** 


### MEDICAL EXAMINER.

DEVOTED TO MEDICINE, SURGERY, AND THE COLLATERAL SCIENCES.

No. 21.] PHILADELPHIA, SATURDAY, MAY 22, 1841. [Vol. IV.

### BIBLIOGRAPHICAL NOTICE.

*Transactions of the Medical Society of the State of New York. Vol. V. Part I.*

THESE Transactions are in general replete with valuable matter—the number before us particularly so. It contains the following papers:—On Inflammatory Fever, by Dr. Ely; on the Nervous System, by Dr. Davis; on Ergot, by Dr. J. B. Beck; on the Ligature of the Femoral and External Iliac Arteries, by Professor Portal, of Palermo; and on Hereditary Diseases, by Dr. Haynes.

Under the vague term inflammatory fever, we are at a loss to understand what fever is described by Dr. Ely, as the symptoms enumerated are merely the common symptoms of fever in general, and no pathology of the disease is offered. If by "true, simple, pure, and uncomplicated INFLAMMATORY FEVER," be meant the ordinary ephemeral fever, the symptoms appear to us to be exaggerated, and an undue severity of treatment enjoined,—in fact, a disease is described which no practitioner here would admit as a simple ephemeral.

Dr. Beck's paper on the abuse of ergot is of great value. Dr. Beck brings strong testimony to the support of the opinion that the peculiar effect of ergot upon the uterus causes a degree of pressure upon the child, which endangers its safety. The general use of it, he thinks, is one of the causes of the great increase in the number of still-born children. To what extent, then, are we justified in using the ergot? Sound practitioners will agree with Dr. Beck, that "in a professional as well as moral point of view, we have no more right to trifle with the life of the child than we have with the life of the mother. When, however, from the nature of the case, it becomes manifest that the life of the mother is in danger, we are not merely justified in using, but it is a positive duty to do so, every means to save her, disregarding every consequence that may result to the child. Now it is for such contingencies that I conceive that ergot ought to be reserved. It should accordingly, I think, never be used except in cases where nature is incompetent to a safe delivery. By too many, it is to be feared, it has been and still is used merely as a *time-saving* agent. Than this, I cannot conceive of any practice more unjustifiable and reprehensible. As a general rule, nature is competent to a safe delivery, and we may rest assured that the best plan is to leave her alone to accomplish the work. Artificial and violent interference, whether it be applied in the shape of instruments or by the use of ergot, cannot but be improper."

